# Risk model and validation of carbapenem-resistant *Klebsiella pneumoniae* infection in patients with cerebrovascular disease in the ICU

**DOI:** 10.1515/med-2023-0774

**Published:** 2023-08-24

**Authors:** Qiuxia Liao, Zhi Feng, Xiaoli Chen

**Affiliations:** Department of Intensive Care Unit, The First Affiliated Hospital of Fujian Medical University, Fuzhou 350005, China; Department of Thoracic Surgery, The First Affiliated Hospital of Fujian Medical University, Fuzhou 350005, China; Department of Intensive Care Unit, National Regional Medical Center, Binhai Campus of the First Affiiliated Hospital, Fujian Medical University, Fuzhou 350212, China

**Keywords:** cerebrovascular disease, carbapenem-resistant *Klebsiella pneumoniae* infection risk model, carbapenem-susceptible *Klebsiella pneumoniae*

## Abstract

Carbapenem-resistant *Klebsiella pneumoniae* (CRKP) is an emerging global epidemic. The intention of this study was to explore the risk model and validation of CRKP infection in patients with cerebrovascular disease in the intensive care unit (ICU). The data of patients with cerebrovascular disease and *Klebsiella pneumoniae* infection were retrospectively collected. The patients were divided into the CRKP group and the carbapenem-susceptible *Klebsiella pneumoniae* (CSKP) group. There were significant differences (*P* < 0.05) between the CRKP group and the CSKP group for many variables. Multivariate binary logistic regression analysis showed that the number of types of antibiotics used, history of glucocorticoid use, and duration of mechanical ventilation before the occurrence of infectious bacteria are the independent risk factors for CRKP infection in patients with cerebrovascular disease in the ICU, and a nomogram risk model was constructed accordingly. The area under the ROC curve of the risk model was 0.868 (95% CI: 0.803–0.934).

## 1 Introduction

Over the past 10 years, with the widespread use of carbapenem antibiotics, the emergence of carbapenem-resistant *Klebsiella pneumoniae* (CRKP) has become a global epidemic, posing a serious threat to human health [1]. According to the results of bacterial resistance monitoring in China in 2020, *Klebsiella pneumoniae* infection ranked the second, and more than eight-fold increase in the resistance rates of *Klebsiella pneumoniae* to meropenem and imipenem was observed from 2.9 and 3% in 2005 to 25 and 26.3%, respectively, in 2018 [2]. CRKP infection is often observed in cerebrovascular patients in the intensive care unit (ICU) and is associated with increased hospitalization costs and mortality rates. Several studies [3,4,5] have found that CRKP infection was associated with previous use of carbapenems, β-lactam, quinolones, and glucocorticoids, and with prolonged hospitalization. However, few studies have analyzed the CRKP infection risk factors and risk models in patients with cerebrovascular disease, and there is a lack of specific scoring criteria for the early identification of these patients. This study retrospectively analyses the data of cerebrovascular patients infected with *Klebsiella pneumoniae*, in order to establish a risk model of CRKP infection in patients with cerebrovascular diseases in the ICU, and to provide a reference for the formulation of subsequent anti-infection programs and prevention and control measures.

## 2 Material and methods

### 2.1 Patients

The data of patients diagnosed with cerebrovascular disease with *Klebsiella pneumoniae* infection admitted to the ICU of the First Affiliated Hospital of Fujian Medical University between January 1, 2018 and September 1, 2020 were collected. The study was approved by the Ethics Review Form for Medical Research and Clinical Technology Application and Ethics committee of the First Affiliated Hospital of Fujian Medical University (MTCA, ECFAH of FMU[2015]084-1). Data acquired must be kept anonymized. Cerebrovascular disease generally refers to cerebrovascular diseases, including cerebral infarction and cerebral hemorrhage, [6] that conform to the International Classification of Diseases (ICD-10). The ICD-10 code includes categories I60–I69. Patients were divided into two groups according to antibiotic susceptibility: the CRKP group and the carbapenem susceptible *Klebsiella pneumoniae* (CSKP) group. Inclusion criteria were as follows: (1) age ≥18 years and (2) the diagnosis of *Klebsiella pneumoniae* was confirmed by a positive culture of the first sputum sample with no other organisms isolated. Exclusion criteria were: (1) patients with multiple pathogens other than *Klebsiella pneumoniae* detected in the first sputum culture and (2) incomplete clinical case data.

### 2.2 Data collection

Basic clinical data of patients were collected, including sex, age, underlying disease, and sputum culture and drug sensitivity results. The following data obtained up to 1 month before the first positive culture were also collected: types of antibiotics used (β-lactam, carbapenems, macrolides, tetracycline, aminoglycosides, lincomycin, polypeptides, polymyxins, sulfonamides, and quinolones), history of hospitalization, history of antibiotic and glucocorticoid use, history of deep vein catheterization, urinary catheterization, gastric tube insertion, tracheal intubation, tracheotomy, mechanical ventilation, hemodialysis, septic shock, and history of vasoactive drug use. The following data, recorded on the day of admission, were also collected: sepsis-related organ failure assessment (SOFA) score, the acute physiology and chronic health evaluation (APACHE II) score, white blood cell count; serum albumin, procalcitonin, and C-reactive protein levels; and the Glasgow Coma Scale Score.

### 2.3 Relevant definitions

Hypertension was defined according to the diagnostic criteria of the Chinese Guidelines for Hypertension Prevention and Treatment [7]. Diabetes was defined according to the diagnostic criteria of the 2022 Chinese Clinical Guidelines for the Prevention and Treatment of Type 2 Diabetes in the Elderly [8]. Hypoproteinemia was defined as a serum albumin level ≤3.5 g/dL [9]. Septic shock was defined as a diagnosis of sepsis combined with persistent hypotension [10]. The SOFA score is based on the degree of organ dysfunction of six organ systems each allocated a score of 0–4 with a maximum of 24 points. The score is calculated on the day of admission and every 24 h using the worse parameters measured during the daily assessment [10]. The APACHE II score is composed of three parts: acute physiological score, age score, and chronic health score, and is a predictor of ICU patient mortality [11]. Nosocomial infection is defined as the infection acquired within 48 h after admission with no evidence of infection on admission or of incubation at the time of admission [12].

### 2.4 Statistical processing

STATA/SE15.0 was used for data analysis. The Shapiro–Wilk test was used to test the normality of the continuous variables. Non-normally distributed continuous variables were reported as median (quartile spacing). The differences between groups were compared by Mann–Whitney *U*-test. The classification variables were expressed as frequency and percentage (%), and the Chi-square test was used to compare the differences between the groups. When the theoretical frequency in the four-cell table was less than five, Fisher’s exact test was used to compare the differences between the groups. Statistically significant variables were screened out through univariate analysis, and the multivariate binary logistic regression stepwise forward method was used to determine the independent risk factors for CRKP infection, which was used to construct the risk model of the column graph. According to the size of the regression coefficients of all the independent variables in the model, the scoring criteria are specified. The Hosmer–Lemeshow goodness of fit test was used to evaluate the calibration of the model. The area under the ROC curve (AUC) was used to evaluate the predictive efficiency of the model. The bootstrap method was used to evaluate the differentiation of the model. Decision curve analysis (DCA) was used to evaluate the clinical value of the model. *P* < 0.05 was considered statistically significant.

## 3 Results

### 3.1 Resistance analysis of CRKP and baseline characteristics of risk models

A total of 112 patients in the ICU with cerebrovascular disease and *Klebsiella pneumoniae* infection were included in the study with 98 male and 14 female participants aged 61.0 ± 16.2 years (range 20–92 years). Cerebrovascular disease included: cerebral hemorrhage, 65 cases; cerebral infarction, 34 cases; cerebral hemorrhage complicated by cerebral infarction, 13 cases. Sixty-seven patients (60%) were infected with CRKP and 45 patients (40%) were infected with CSKP. Drug resistance analysis of CRKP showed that the drug resistance rates of cefoperazone/tazobactam, piperacillin/tazobactam, ceftazidime, and ceftriaxone were 98.51, 97.01, 98.51, and 98.51%, respectively. The drug resistance rate to cefepime, aztreonam, meropenem, and imipenem was up to 100%. The resistance rates to amikacin and co-trimoxazole were 77.61 and 79.10%, respectively. The drug resistance rate to tigecycline and polymyxin was low, and the antibacterial activity was good *in vitro* (Table 1).

**Table 1:** Antibiotic susceptibility testing results of CRKP

Antibiotics	Number of strains (*n* = 67)	Rate of drug resistance (%)
Cefoperazone/tazobactam	66	98.51
Piperacillin/tazobactam	65	97.01
Ceftazidime	66	98.51
Ceftriaxone	66	98.51
Cefepime	67	100.00
Ammonia aztreonam	67	100.00
Imipenem	67	100.00
Meropenem	67	100.00
Amikacin	52	77.61
Tobramycin	57	85.07
Ciprofloxacin	64	95.52
Levofloxacin	62	92.54
Co-trimoxazole	53	79.10
Tigecycline	5	7.46
Polymyxins	0	0.00

There were significant differences (*P* < 0.05) between the CRKP group and the CSKP group for the following variables: history of hospitalization; history of hydrocarbon-enzyme antibiotics, β-lactam, antifungal, combined treatment with multiple antibiotics, and glucocorticoid use; history of deep vein catheterization; duration of mechanical ventilation before infection; duration of antibiotic use; and the number of types of antibiotics used. There were no significant differences between the two groups for sex, age, hypertension, diabetes, septic shock, hypoproteinemia, APACHE II score, SOFA score, Glasgow Coma Scale score, white blood cell count, procalcitonin, and C-reactive protein (*P* > 0.05, Table 2).

**Table 2:** Baseline characteristics of CSKP and CRKP infection groups in patients with cerebrovascular disease in ICU

Variable (*n* = 112)	CSKP (*n* = 45)	CRKP (*n* = 67)	*Z*/*χ* ^2^	*P*
Sex			0.90	0.344
Female	4 (9)	10 (15)		
Male	41 (91)	57 (85)		
Age (years)	59 (52, 70)	63 (51, 76)	−1.02	0.307
Age ≥60 years			1.27	0.259
No	23 (51)	27 (40)		
Yes	22 (49)	40 (60)		
Hypertension			1.39	0.239
No	19 (42)	21 (31)		
Yes	26 (58)	46 (69)		
Diabetes			1.00	0.317
No	36 (80)	48 (72)		
Yes	9 (20)	19 (28)		
Septic shock			#	0.082
No	44 (98)	59 (88)		
Yes	1 (2)	8 (12)		
Hypoproteinemia			3.01	0.083
No	38 (84)	47 (70)		
Yes	7 (16)	20 (30)		
History of hospitalization			5.57	0.018
No	27 (60)	25 (37)		
Yes	18 (40)	42 (63)		
History of hydrocarbon-enzyme antibiotics			13.52	<0.001
No	37 (82)	32 (48)		
Yes	8 (18)	35 (52)		
History of β-lactam antibiotics			9.07	0.003
No	24 (53)	17 (25)		
Yes	21 (47)	50 (75)		
History of antifungal drug use			#	<0.001
No	45 (100)	48 (72)		
Yes	0 (0)	19 (28)		
History of combined treatment with multiple antibiotics			16.4	<0.001
No	41 (91)	37 (55)		
Yes	4 (9)	30 (45)		
History of glucocorticoid use			5.51	0.028
No	41 (91)	49 (73)		
Yes	4 (9)	18 (27)		
History of deep vein catheterization			9.50	0.002
No	38 (84)	38 (57)		
Yes	7 (16)	29 (43)		
Tracheal intubation				
No	10 (22)	17 (25)	0.15	0.702
Yes	35 (78)	50 (75)		
Tracheotomy				
No	39 (87)	55 (82)	0.42	0.518
Yes	6 (13)	12 (18)		
Hemodialysis				
No	43 (96)	57 (85)	#	0.119
Yes	2 (4)	10 (15)		
Hospital acquired infection			23.82	<0.001
No	14 (31)	0 (0)		
Yes	31 (69)	67 (100)		
SOFA score	5 (4, 6)	5 (4, 7)	−0.18	0.857
APACHE II score	17 (15, 19)	17 (13, 21)	−0.82	0.412
Duration of antibiotic use (days)	1 (0, 5)	11 (5, 17)	−5.70	<0.001
Number of types of antibiotics used	1 (0, 1)	2 (1, 3)	−5.96	<0.001
Duration of mechanical ventilation before infection (days)	1 (0, 1)	3 (1, 7)	−3.76	<0.001
GCS Score	5 (5, 7)	6 (4, 9)	−1.20	0.229
White blood cell count (10^9^/L)	11.34 (8.78, 14.6)	11.8 (8.22, 15.18)	−0.11	0.915
Procalcitonin (ng/mL)	0.39 (0.12, 2.42)	0.47 (0.12, 2.38)	−0.46	0.646
C-reactive protein (mg/L)	75.52 (33.67, 90.00)	59.41 (18.40, 90.00)	1.36	0.174

Abbreviations: ICU: intensive care unit; CRKP: carbapenem-resistant Klebsiella pneumoniae; CSKP: carbapenem-susceptible Klebsiella pneumoniae; SOFA: sepsis-related organ failure assessment; APACHE II: acute physiology and chronic health evaluation; Continuous variables that do not conform to normal distribution represented by (Median [Inter Quartile Range]) were compared using the *Z* test; The classification variable was represented by [*n* (%)], and the variables were compared using the *χ* ^2^ test. #: the Fisher exact test was used.

### 3.2 Multivariate binary logistic regression analysis of CRKP infection in patients with cerebrovascular disease in the ICU

With the occurrence of CRKP infection as the dependent variable, variables with *P* < 0.05 in the univariate analysis were set as the independent variables. These included history of hospitalization; history of hydrocarbon-enzyme antibiotics, β-lactam antibiotics, antifungal drugs, combined treatment with multiple antibiotics, and glucocorticoids in the previous month; history of deep vein catheterization; duration of mechanical ventilation before infection; and duration of antibiotic use and number of types of antibiotics use. Multivariate binary logistic regression was used for stepwise forward analysis. Factors with *P* < 0.05 were included in the equation, indicating number of types of antibiotics use (OR: 3.419, 95% CI: 1.708–6.844). History of glucocorticoid use (OR: 3.985, 95% CI: 1.097–14.475) and duration of mechanical ventilation before infection (OR: 1.203, 95% CI: 1.003–1.442) were independent risk factors for CRKP infection in patients with cerebrovascular disease in the ICU. Model equations for: *P* = 1/[1 + exp [–(‒2.055 + 1.229*X*1 + 1.383*X*2 + 0.185*X*3)]] (Table 3).

**Table 3:** Multivariate logistic regression analysis of carbapenem-resistant Klebsiella pneumoniae infection in patients with cerebrovascular disease in the ICU

Independent variable	OR	95% CI	*P*
Number of types of antibiotics used (*X*1)	3.419	1.708–6.844	0.001
History of glucocorticoid use (*X*2)	3.985	1.097–14.475	0.036
Duration of mechanical ventilation before infection (days) (*X*3)	1.203	1.003–1.442	0.046

ICU: intensive care unit; CRKP: carbapenem-resistant Klebsiella pneumoniae; OR: odds ratio; CI: confidence interval; *X*1, *X*2, and *X*3 refer to the forward step method of multivariable logistic regression analysis, and factors with *P* < 0.05 were included in the model.

### 3.3 Intuitive nomogram of a risk model

**Figure 1:**
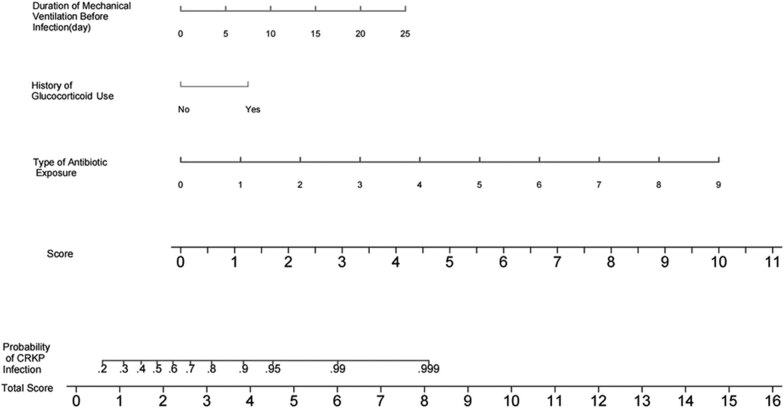
Visual nomogram for predicting CRKP infection in patients with cerebrovascular disease in the ICU. ICU: Intensive care unit; CRKP: Carbapenem-resistant Klebsiella pneumoniae.

The independent risk factors screened by multiple logistic regression were used to construct a risk model of CRKP infection in patients with cerebrovascular disease in the ICU, and a nomogram map was drawn. The corresponding scores were calculated by the regression coefficient of each variable: 4.2 points for duration of mechanical ventilation before infection, 1.2 points for history of glucocorticoid use, 10.0 points for number of types of antibiotics used, and 0–15.4 points for model scores. The individual scores of each variable were added together to obtain the total score, which corresponds to the probability of predicting CRKP infection in patients with cerebrovascular disease in the ICU. The nomogram indicated that the total score was greater than 3, and the probability of infection with CRKP in patients with cerebrovascular disease in the ICU was 80% (Figure 1).

### 3.4 Internal validation of the risk model

**Figure 2:**
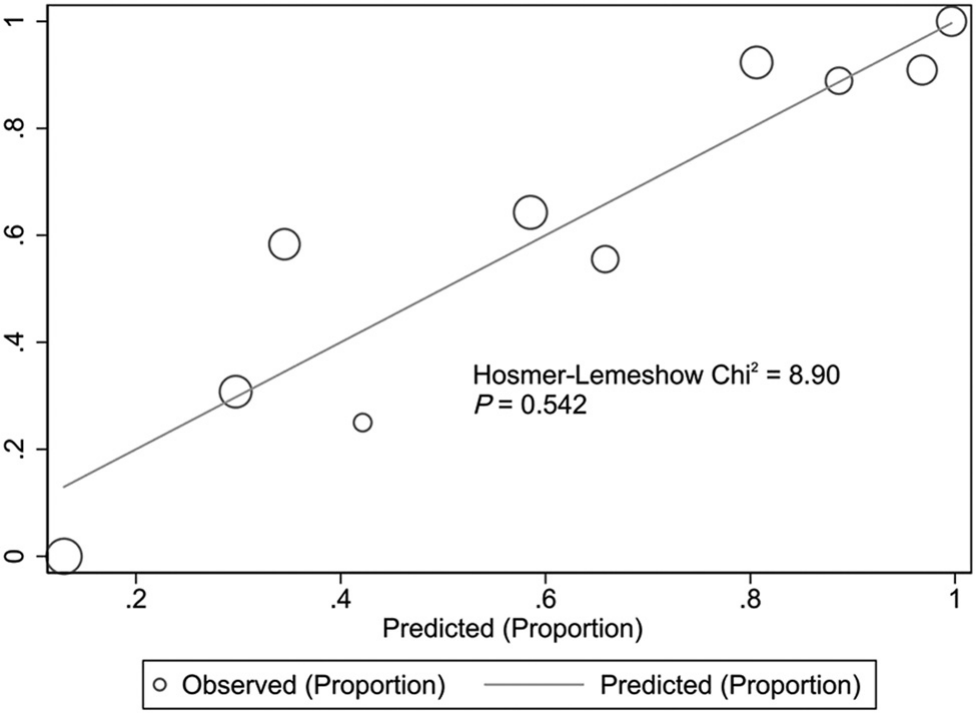
Hosmer–Lemeshow diagram for the risk model.

**Figure 3:**
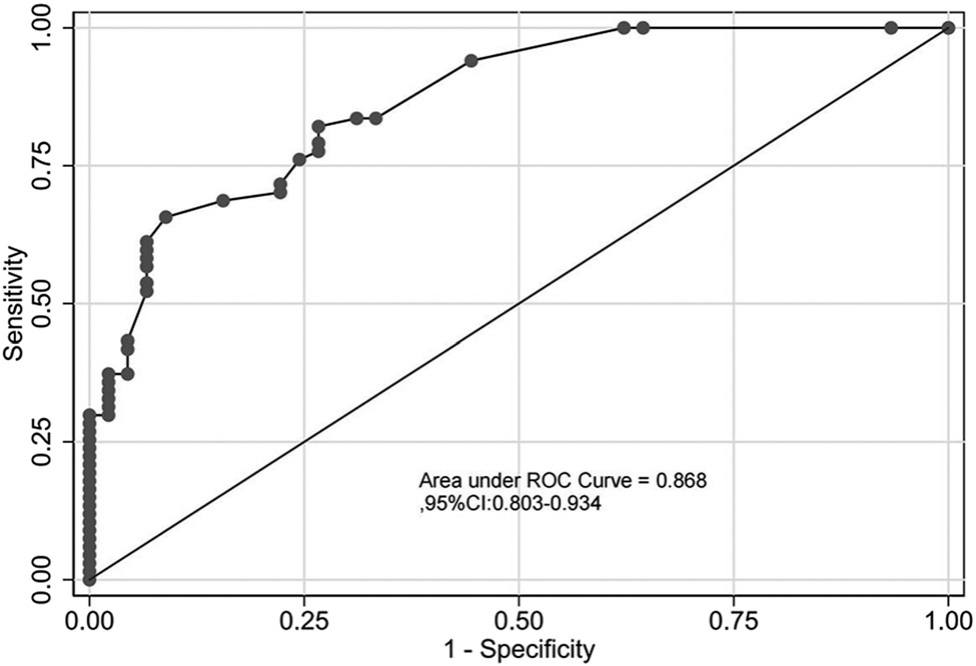
ROC diagram of the risk model.

**Figure 4:**
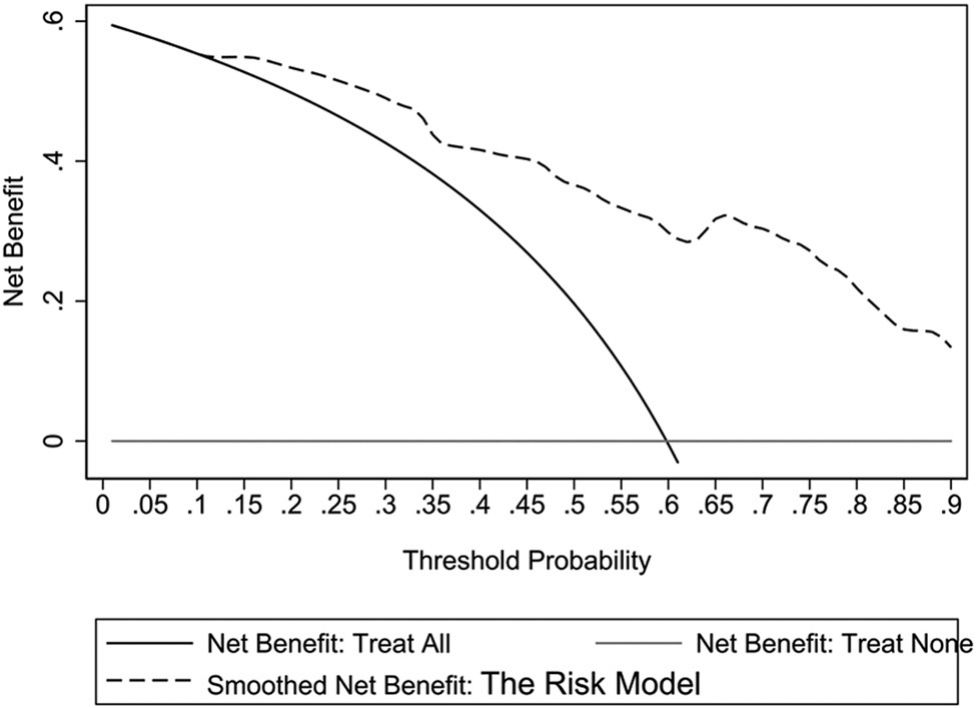
DCA diagram of the risk model.

A Chi-square of 8.90, and a *P*-value = 0.542 was obtained in the risk model using the Hosmer–Lemeshow goodness of fit test, indicating a good fit of the model (Figure 2). The AUC of the risk model was 0.868 (95% CI: 0.803–0.934), indicating that the prediction efficiency of the model was good (Figure 3). The Bootstrap method was used for internal verification. The number of repetitions was 1,000, and the AUC was 0.868 (95% CI: 0.804–0.932), indicating good differentiation and repeatability of the model. The DCA decision curve was drawn and showed that when the prediction probability was greater than 0.6, the model had the greatest clinical benefit, indicating that the risk model had high clinical practical value (Figure 4).

## 4 Discussion

CRKP can produce multiple carbapenemases and/or broad-spectrum beta lactamases, accompanied by membrane pore-protein loss and efflux pump overexpression, ultimately leading to the generation of multidrug-resistant, extensively drug-resistant, and pan-drug-resistant bacteria [13]. CRKP resistance mechanism mainly includes [14]: (1) CRKP produces carbapenemase; (2) CRKP strain yields high AmpC enzyme or ultra-broad spectrum β lactamase (ESBL); (3) Loss or decreased expression of CRKP outer membrane protein prevents antibiotics from entering bacteria and lead to drug resistance; (4) The high expression can be found in the CRKP efflux pump, bacteria can transfer antibiotics from the bacteria outside the bacteria through the efflux pump, so as to achieve the effect of drug resistance; (5) Loss/decrease of PBP2 at the high affinity binding site of CRKP carbapenems or decrease of affinity is also one of the reasons for drug resistance. These multiple resistance mechanisms can exist alone or together to show increased resistance to carbapenem antibiotics and cross-resistance to other antibiotics. The drug sensitivity of CRKP was analyzed showing a CRKP resistance rate of up to over 90% to a variety of antibiotics, including cefoperazone/tazobactam, piperacillin/tazobactam, ceftazidime, ceftriaxone, cefepime, amtrannan, meropenem, imipenem, levofloxacin and ciprofloxacin. CRKP was sensitive only to tigecycline and polymyxin. Antibiotic resistance of CRKP poses a considerable challenge to clinical treatment [15]. Patients with cerebrovascular disease in the ICU are at high risk of CRKP infection due to severe illness, poor immunity, long-term hospitalization, and antibiotic use. Infection is associated with increased hospitalization costs and mortality rates.

The results of this study showed that the use of carbapenem antibiotics and β-lactam antibiotics was associated with CRKP infection in patients with cerebrovascular disease in the ICU, which was consistent with the results reported by Dai et al. [3] and Tumbarello et al. [16]. The results of this study suggested that the type of antibiotic exposure was an independent risk factor for CRKP infection in patients with cerebrovascular disease in the ICU. This was consistent with the findings by Dai et al. [3] that history of antibiotic use was a risk factor for CRKP infection. Together, these results suggest that CRKP production is associated with the history of clinical antibiotic use, and reducing unnecessary and inappropriate antibiotic use can effectively prevent CRKP infection.

In this study, the duration of mechanical ventilation before infection was correlated with CRKP infection. First of all, patients with cerebrovascular disease in ICU requiring mechanical ventilation tend to be seriously ill. Patients with poor immunity, impaired brain function, analgesic and sedative drugs, and low airway clearance ability have increased susceptibility to CRKP [17,18,19]. Second, patients with mechanical ventilation often have invasive procedures such as tracheal intubation and tracheotomy, which increase the chance of CRKP invasion [20]. Wang et al. [21] also found that long-term mechanical ventilation increased the resistance of *Klebsiella pneumoniae* to carbapenem antibiotics. Early removal from the ventilator-assisted breathing and reducing the time of mechanical ventilation can also reduce the production of CRKP. This study visualized the scoring criteria by drawing a Nomogram. The probability of CRKP infection in patients with cerebrovascular disease in ICU can be estimated according to the score, which is of high clinical convenience and can be used for targeted intervention in advance.

Glucocorticoids have immunosuppressive effects and induce cellular immune deficiency, which may increase the susceptibility of the host to bacteria [22]. The results of this study indicated that the history of glucocorticoid use was a risk factor for CRKP in patients with cerebrovascular disease in the ICU, showing that a strict control of glucocorticoid use was important. If glucocorticoid therapy is required, the dose of glucocorticoid should be reduced as much as possible and the duration of glucocorticoid use should be shortened to reduce the risk of infection.

The following three risk factors were included in the risk model: number of types of antibiotics used, history of glucocorticoid use, and duration of mechanical ventilation before infection. A large AUC curve area of 0.868 was obtained, indicating the strong discrimination ability of the model, and the results of the Hosmer–Lemeshow test of the model showed goodness of fit with the actual observed values matching the expected values of the model. These results suggested that the risk model could effectively predict the risk of CRKP infection in patients with cerebrovascular disease in the ICU. Yang et al. [23] found that age, male sex, cardiovascular disease, hospitalization, recent stay in the ICU, indwelling catheter, history of mechanical ventilation, recent use of β-lactams, and fourth-generation cephalosporins, and carbapenems were independent risk factors for CRKP infection. The authors built a CRKP infection prediction model based on these data with an AUC of 0.899, which was comparable to that in our study. However, many factors were included in their model, and there was no visual scoring standard so that its use in clinical practice was limited. The model established in this study included few factors and the scoring criteria were visualized by drawing a nomogram. The score ranged from 0 to 14.8, and the probability of CRKP infection in patients with cerebrovascular disease in the ICU could be estimated according to the score. The present model is easily applicable in clinical practice and can be used for targeted intervention in advance to reduce the blindness of medical staff in the treatment and for effective and reasonable use of antibiotics. After the patient is admitted to the ICU, even if the etiological culture is performed immediately, it will take a long time to get the results of the etiological culture. In order to reduce the occurrence of CRKP and reduce the length of hospital stay and patient mortality rates, the clinician can score the patient according to the risk model, predict the probability of CRKP infection, and treat high-risk patients with effective antibiotics as soon as possible.

However, this study was a single-center retrospective study with limited sample size and only internal validation was performed. This study mainly focused on patients with cerebrovascular disease complicated with CRKP infection, and did not target general patients with CRKP. Further multi-center and large sample prospective studies are required to validate the risk model developed in this study.

## 5 Conclusion

Our findings revealed that the number of types of antibiotics used, history of glucocorticoid use, and duration of mechanical ventilation before infection were the independent risk factors for CRKP infection in patients with cerebrovascular disease in the ICU. This risk model may be used as a reference to develop targeted anti-infective treatment plans and preventive measures.
